# Osteoplant acts on stem cells derived from peripheral blood

**DOI:** 10.4103/0972-124X.65429

**Published:** 2010

**Authors:** Vincenzo Sollazzo, Annalisa Palmieri, Ambra Girardi, Ilaria Zollino, Giorgio Brunelli, Giuseppe Spinelli, Francesco Carinci

**Affiliations:** *Orthopedic Clinic, Corso Giovecca 203, 44100 Ferrara, Italy*; 1 *Department of Maxillofacial Surgery, University of Ferrara, Corso Giovecca 203, 44100 Ferrara, Italy*; 2 *Institute of Histology, Embryology and Applied Biology, University of Bologna, Via Belmeloro 8, 40100 Bologna, Italy*; 3 *Department of Maxillofacial Surgery, Carreggi Hospital, Via Pieraccini 18, 50139, Firenze, Italy*

**Keywords:** Gene expression, stem cell, osteoplant, bone

## Abstract

**Objectives:**

The osteoplant is an equine, flexible, heterologous, deantigenic, cortical, and spongy bone tissue, totally reabsorbable, used for implantation of bone tissue, to restore skeletal, even weight-bearing structures. However, how the osteoplant alters osteoblast activity to promote bone formation is poorly understood.

**Materials and Methods:**

To study how the osteoplant induces osteoblast differentiation in mesenchymal stem cells, the expression levels of bone-related genes, and mesenchymal stem cell markers are analyzed, using real time Reverse Transcription-Polymerase Chain Reaction (RT-PCR).

**Results:**

The osteoplant causes induction of osteoblast transcriptional factors such as osterix (RUNX2), and of bone-related genes such as osteopontin (SPP1) and osteocalcin (BGLAP). In contrast the expression of ENG (CD105) is significantly decreased in stem cells treated with osteoplant, with respect to untreated cells, indicating the differentiation effect of this biomaterial on stem cells.

**Conclusion:**

The obtained results can be relevant to better understand the molecular mechanism of bone regeneration and as a model for comparing other materials with similar clinical effects.

## INTRODUCTION

Autogenous iliac crest bone graft has been the ‘gold standard’ for spinal fusion and maxillary sinus augmentation. However, bone graft harvest may lead to complications, such as, chronic pain, numbness, and poor cosmesis.[1] Bone grafting procedures are undergoing a major shift from autologous and allogeneic bone grafts to synthetic bone graft substitutes.[2] Biomaterials used in bone regeneration are designed to be gradually resorbed by the osteoclast and replaced by new bone formed through osteoblastic activity.[3] Large bone defects still represent a major problem in orthopedics and maxillofacial surgery. Traditional bone-repair treatments can be divided into two groups: the bone transport (Ilizarov technology) and the graft transplant (autologous or allogeneic bone grafts). In our study we used the osteoplant (Bioteck SRL, Vicenza, Italy), which is an equine, flexible, heterologous, deantigenic, cortical, and spongy bone tissue, totally reabsorbable, used for implantation of bone tissue, to restore skeletal and even weight-bearing structures.

In a previous study, Perrotti *et al*.[4] demonstrated that equine bone was conducive to the osteoblast and osteoclast and for this reason well reabsorbable. They evaluated osteoclast formation, attachment, and resorptive activity on the equine bone. The resorption lacunae were observed on the equine bone. In addition the collagen was detected in the resorption pits generated on the equine spongy bone, with the formation of the characteristic actin ring. This evidence demonstrated that this bone substitute material was actively resorbed by human osteoclasts.

The antigenic component of the animal spongy bone scaffold is selectively eliminated throughout an innovative biophysical and biochemical process. These processes allow the removal of all the proteins inside the animal bone that are genetically different, thus being able to activate an immunologic reaction against the graft. The bony scaffold obtained in this way is compatible, osteoconductive, and useful to fill bone defects in orthopedic, maxillofacial, and dental surgeries. The osteoplant, once hydrated, is flexible and therefore precisely adaptable to the defect that needs to be filled. The mechanism by which the osteoplant seems to induce the reabsorption and the substitution of the graft with the new bone starts from the activation of the osteoclast. Osteoclast activation then induces bone formation, resulting in a complete substitution of the scaffold with the regenerated bone.[4]

As few reports analyze the effects of the osteoplant on stem cells[5,6] and none focus on the genetic effects, the expression of genes related to osteoblast differentiation have been analyzed using cultures of stem cells derived from peripheral blood (PB-hMSCs) treated with osteoplant.

To investigate the osteogenic differentiation of PB-hMSCs, the quantitative expression of the mRNA of specific genes, such as, transcriptional factors (RUNX2 and SP7), bone-related genes (SPP1, COL1A1, COL3A1, BGLAP, ALPL, and FOSL1), and mesenchymal stem cell markers (ENG) were examined, by means of real time Reverse Transcription-Polymerase Chain Reaction (RT-PCR).

## MATERIALS AND METHODS

### a) Stem preparation

PB-hMSCs were obtained for gradient centrifugation from the peripheral blood of healthy anonymous volunteers, using the Acuspin System-Histopaque 1077 (Sigma Aldrich, Inc., St Louis, Mo, USA). First, 30 ml of heparinizated peripheral blood was added to the Acuspin System-Histopaque 1077 tube and centrifugated at 1000 × g for 10 minutes. After centrifugation the interface containing mononuclear cells was transferred to another tube, washed with PBS, and centrifugated at 250 × g for 10 minutes. The enriched mononuclear pellets was resuspended in 10 ml of Alphamem medium (Sigma Aldrich, Inc., St Louis, Mo, USA) supplemented with antibiotics (Penicillin 100 U/ml and Streptomycin 100 micrograms/ml - Sigma, Chemical Co., St Louis, Mo, USA) and amino acids (L-Glutamine - Sigma, Chemical Co., St Louis, Mo, USA). The cells were maintained in a 5% CO_2_ humidified atmosphere at 37°C. The medium was changed after 24 hours. PB-hMSC were selected for adhesivity and characterized for staminality by immunoflorescence.

### b) Immunofluorescence

Cells were washed with PBS for three times and fixed with cold methanol for five minutes at room temperature. After washing with PBS, the cells were blocked with bovine albumin 3% (Sigma Aldrich, Inc., St Louis, Mo, USA) for 30 minutes at room temperature. The cells were incubated overnight sequentially at 4°C with primary antibodies raised against CD105 1:200, mouse (BD Biosciences, San Jose, CA, USA), CD73 1:200, mouse (Santa Cruz Biotecnology, Inc., Santa Cruz, CA, USA), CD90 1:200, mouse (Santa Cruz Biotecnology, Inc., Santa Cruz, CA, USA), and CD34 1:200, mouse (Santa Cruz Biotecnology, Inc., Santa Cruz, CA, USA). They were washed with PBS and incubated for one hour at room temperature with secondary antibody Rodamine conjugated goat anti-mouse 1:200 (Santa Cruz Biotecnology, Inc., Santa Cruz, CA, USA). Subsequently, the cells were mounted with the Vectashield Mounting Medium with 4’-6-Diamidino-2-phenylindole (DAPI) (Vector Laboratories, Inc., Burlingame, CA, USA) and observed under a fluorescence microscope (Eclipse TE 2000-E, Nikon Instruments S.p.a., Florence, Italy).

### c) Cell culture

PB-hMSCs, at second passage, were cultured in an Alphamem medium (Sigma Aldrich, Inc., St Louis, Mo, USA) supplemented with 10% fetal calf serum, antibiotics (Penicillin 100 U/ml and Streptomycin 100 micrograms/ml - Sigma Aldrich, Inc., St Louis, Mo, USA) and amino acids (L-Glutamine - Sigma Aldrich, Inc., St Louis, Mo, USA).

The cultures were maintained in 5% CO_2_ humidified atmosphere at 37°C. For the assay, the cells were collected and seeded at a density of 1 × 10^5^ cells/ml into 9 cm^2^ (3 ml) wells by using 0.1% trypsin, 0.02% EDTA in Ca++, and Mg – free Eagle’s buffer for cell release.

One set of wells was added with the osteoplant (Bioteck SRL, Vicenza, Italy) at a concentration of 10 mg/ml. Another set of wells containing untreated cells was used as control. The medium was changed every three days.

After seven days, when the cultures were sub-confluent, the cells were processed for RNA extraction.

### d) RNA processing

Reverse transcription for cDNA was performed directly from the cultured cell lysate using the TaqMAn Gene Expression Cells-to-Ct Kit (Ambion Inc., Austin, TX, USA), following the manufacturer’s instructions. Briefly, the cultured cells were lysed with a lysis buffer and the RNA released in this solution. Cell lysates were reverse transcribed to cDNA using the RT Enzyme Mix and appropriate RT buffer (Ambion Inc., Austin, TX, USA).

Finally the cDNA was amplified by real-time PCR using the included TaqMan Gene Expression Master Mix and the specific assay designed for the investigated genes.

### e) Real time PCR

Expression was quantified using real time RT-PCR. The gene expression levels were normalized to the expression of the housekeeping gene, RPL13A, and were expressed as fold changes, relative to the expression of the untreated PB-hMSCs. Quantification was done using the delta / delta calculation method.[7]

Primers and probes for the selected genes were designed with Primer Express software (Applied Biosystems) and are listed in Table 1.

**Table 1:** Primer and probes used in real time PCR

Gene symbol	Osteopontin	Primer sequence (5’>3’)	Probe sequence (5’>3’)
SPP1	Osteopontin	F-GCCAGTTGCAGCCTTCTCA	CCAAACGCCGACCAAGGAAAACTCAC
		R-AAAAGCAAATCACTGCAATTCTCA	
COL1A1	Collagen type I alpha1	F-TAGGGTCTAGACATGTTCAGCTTTGT	CCTCTTAGCGGCCACCGCCCT
		R-GTGATTGGTGGGATGTCTTCGT	
RUNX2	Runt-related transcription factor 2	F-TCTACCACCCCGCTGTCTTC	ACTGGGCTTCCTGCCATCACCGA
		R-TGGCAGTGTCATCATCTGAAATG	
ALPL	Alkaline phospatase	F-CCGTGGCAACTCTATCTTTGG	CCATGCTGAGTGACACAGACAAGAAGCC
		R-CAGGCCCATTGCCATACAG	
COL3A1	Collagen, type III, alpha 1	F-CCCACTATTATTTTGGCACAACAG	ATGTTCCCATCTTGGTCAGTCCTATGCG
		R-AACGGATCCTGAGTCACAGACA	
BGLAP	Osteocalcin	F-CCCTCCTGCTTGGACACAAA	CCTTTGCTGGACTCTGCACCGCTG
		R-CACACTCCTCGCCCTATTGG	
CD105	Endoglin	F-TCATCACCACAGCGGAAAAA	TGCACTGCCTCAACATGGACAGCCT
		R-GGTAGAGGCCCAGCTGGAA	
FOSL1	FOS-like antigen 1	F-CGCGAGCGGAACAAGCT	ACTTCCTGCAGGCGGAGACTGACAAAC
		R-GCAGCCCAGATTTCTCATCTTC	
SP7	Osterix	F-ACTCACACCCGGGAGAAGAA	TCACCTGCCTGCTCTTGCTCCAAGC
RPL13A	Ribosomal protein L13	F-AAAGCGGATGGTGGTTCCT	CTGCCCTCAAGGTCGTGCGTCTG

All PCR reactions were performed in 20 μl volume using the ABI PRISM 7500 (Applied Biosystems, Foster City, CA, USA). Each reaction contained 10 μl of 2 X TaqMan universal PCR master mix (Applied Biosystems, Foster City, CA, USA), 400 nM concentration of each primer, and 200 nM of the probe, and cDNA. The amplification of the profile was initiated by a 10 minute incubation, at 95°C, followed by a two-step amplification of 15 seconds at 95°C, and 60 seconds at 60°C, for 40 cycles. All experiments were performed, including non-template controls, to exclude reagent contamination. PCRs were performed with two biological replicates.

## RESULTS

**Figure 1:**
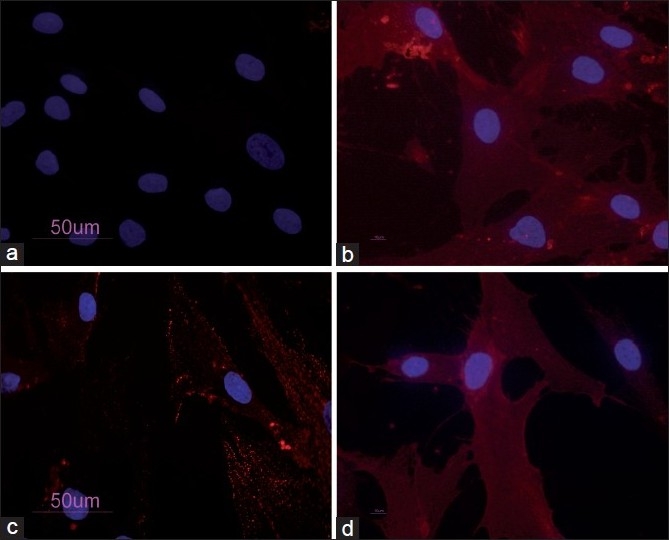
PB-hMSCs by indirect immunofluorescence (Rodamine). Cultured cells were positive for the mesenchymal stem cell marker CD73 (a), CD90 (b), CD105 (c) and negative for the hematopoietic markers CD34 (d). Nucleuses were stained with DAPI. Original magnification ×40

The PB-hMSCs were characterized by immunofluorescence. The cell surfaces were positive for mesenchymal stem cell markers, CD105, CD90, and CD73, and negative for the marker of the hematopoietic origin, CD34 [Figure 1].

Transcriptional expressions of several osteoblast-related genes (RUNX2, SP7, SPP1, COLIA1, COL3A1, BGLAP, ALPL, and FOSL1) and mesenchymal stem cell markers (ENG) were examined after seven days of treatment with osteoplant.

The results of real-time PCR showed that after one week of treatment, compared to the control cells, the osteogenic transcription factors RUNX2 and two bone-related genes BGLAP and SPP1 were upregulated.

**Figure 2:**
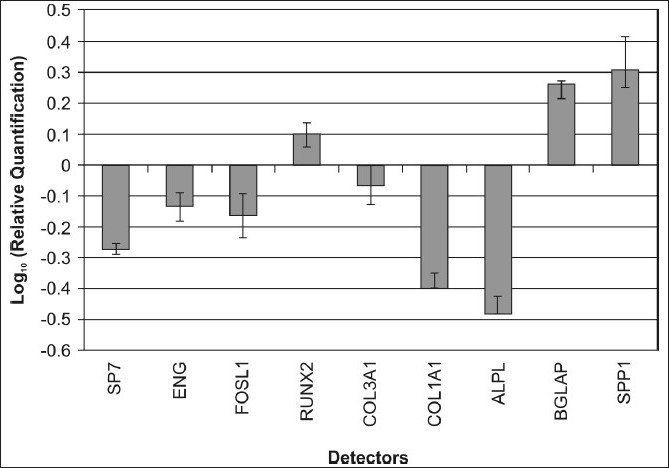
Gene expression analysis of PB-hMSCs after 7 days of treatment with osteoplant

COL1A1 and COL3A, SP7, and FOSL1 and ALPL were decreased in the presence of the osteoplant on day seven, similar to the stem cell marker ENG [Figure 2].

## DISCUSSION

Although autogenous bone grafting continues to be considered the gold standard for sinus grafting and spinal fusion,[8] it is associated with an unacceptably high incidence of morbidity.[9] Chronic iliac crest bone graft harvest site pain and discomfort is reported by a significant percentage of patients undergoing this procedure, more than three years following surgery, and these complications are associated with worse patient-reported disability.[1] Furthermore, operative time and length of hospitalization are often increased.[9] In order for a graft substitute to replicate the optimal bone healing properties of an autogenous graft, three essential elements must be present: scaffolding for osteoconduction, growth factors for osteoinduction, and progenitor cells for osteogenesis. A composite graft that combines a synthetic scaffold with osteoprogenitor cells from a bone marrow aspirate (BMA) may potentially deliver the advantages of autogenous bone grafts without procurement morbidity.[8] Tissue engineering for bone grafting may emerge as an alternative for autogenous bone grafts.[10]

Tissue engineering of the bone entails the successful interplay between osteoinductive factors, osteogenic cells, their extracellular environment, and an osteoconductive biomaterial scaffold.

Research in regenerative medicine is developing at a significantly rapid pace. Cell-based bone and cartilage replacement is an evolving therapy aimed at the treatment of patients who suffer from limb amputation, damaged tissues, and various bone and cartilage-related disorders, as also dental and maxillofacial reconstructive surgery. Stem cells isolated from multiple sources have been finding widespread use in the advancement in the field of tissue repair.[11] Bone regeneration for the defects in revision surgery of joint replacement is an increasingly important issue. To repair bone defects, bone cell activation by growth factors, using a synthetic resorbable scaffold is a useful and safe option.[12] Synthetic and biological materials are increasingly used to provide temporary or permanent scaffolds for bone regeneration.[13] Bone regeneration by autologous cell transplantation in combination with a biodegradable scaffold is one of the most promising techniques being developed in craniofacial and orthopedic surgeries.[14] Tissue engineering approaches attempt to create tissue replacement by culturing autologous cells into three-dimensional matrices that facilitate cell progenitor migration, proliferation, and differentiation.[15]

Maxillary sinus augmentation is frequently necessary before placement of dental implants in the posterior maxilla. Besides the autogenous bone graft, various bone substitutes have been used, with favorable results.[16] In a retrospective study of over 14 years, Ewers has shown that the sinus lift procedure, with grafting of the sinus floor and subsequent implant placement is a proven method and he has also shown that the marine-derived HA material, ACA, in a mixture with approximately 10% autogenous collector bone and blood or platelet rich plasma, is able to enhance enough new bone in six months, to allow implant osseointegration after six more months, with a high implant survival rate.[8]

Among the biomaterials used for sinus elevation procedures, xenografts are very popular.[17] They are highly attractive because they carry a small risk of contamination from infectious diseases, do not compromise the patients remaining tissues, and may have the correct structure as the component being replaced.[17] Due to their chemical – physical characteristics similar to those of the human bone, these natural materials show great osteoconductive properties.[18] In addition, the xenograft for bone substitution appears to activate osteoclastogenesis. Oteoclast activation promotes both scaffold absorption and substitution of the scaffold with a new bone simultaneously. Perrotti *et al*.[4] demonstrated that osteoclasts resorbed equine spongy bone.

In this study we focus our interest on a new equine, flexible, heterologous, deantigenic, cortical, and spongy bone tissue, osteoplant.

In order to get a better insight into how the osteoplant acts on PB-hMSCs, changes in expression of bone-related marker genes (RUNX2, SP7, SPP1, COLIA1, COL3A1, BGLAP, ALPL, and FOSL1) and mesenchymal stem cells markers (ENG) were investigated by real-time RT-PCR.

In our study, the mesenchymal stem cells from peripheral blood were isolated and characterized by morphology and immunophenotype. Isolated PB-hMSCs showed fibroblast-like morphology and were positive for MSC surface molecules (CD90, CD105, CD73) and negative for markers of hematopoietic progenitors (CD34).

After a seven-day treatment with the osteoplant the expression levels of osteodifferentiation genes were measured by relative quantification methods using real-time RT-PCR.

Two transcriptional factors had an opposite expression. RUNX2 was upregulated in treated PB-hMSCs, with respect to control, while SP7, was downregulated.

RUNX2 is a key transcriptional modulator of osteoblast differentiation that plays a fundamental role in osteoblast maturation and homeostasis. RUNX2-null mice despite normal skeletal patterning have no osteoblasts and consequently bone tissue. RUNX2 at the early stage of embryogenesis determines the osteoblast lineage from multipotent mesenchymal stem cells.[19] Thus we demonstrate that osteoplant increases the activity of the RUNX2 gene, which is a key point in osteodifferentiation.

SP7 is a zinc finger transcriptional factor that regulates bone formation and osteoblast differentiation *in vitro* and *in vivo* and is downstream of RUNX2; however, in our experimental model, SP7 expression was downregulated during osteogenic induction, probably because this gene regulates the later stages of osteoblast differentiation and bone development.[20] This result is not in contrast with those previously reported as we investigated stem cells treated for seven days.

Also BGLAP (a bone specific protein involved in mineralization and bone resorption) and SPP1 (an acid phosphoprotein involved in regulation of bone mineralization) were expressed early in osteogenic progression of PB-hMSCs treated with osteoplant.[21] This is a further demonstration that osteoplant induces stem differentiation.

SPP1 encodes osteopontin, which is a phosphoglycoprotein of the bone matrix and it is the most representative noncollagenic component of the extracellular bone matrix.[13] Osteopontin is actively involved in bone resorbitive processes directly carried out by ostoclasts.[14] Osteopontin produced by the osteoblasts, shows a high affinity to the molecules of hydroxylapatite in the extracellular matrix and is a chemo-attractant to the osteoclasts.[22] Osteoblastic bone formation is associated with osteoclastic bone resorption during the bone remodeling process. For this reason the surface of the implanted material should be conducive to osteoblast and osteoclast activity.[23] Thus an increase of Osteopontin facilitates osteoclast activity.

ENG (CD105), a surface marker used to define a bone marrow stromal cell population, capable of multilineage differentiation,[24] was down expressed in treated PB-hMSCs, with respect to control, at seven days, indicating the differentiation effect of this biomaterial on stem cells. The disappearance of the CD105 antigen during osteogenesis suggests that this protein, similar to others in the TFG-β superfamily, is involved in the regulation of osteogenesis.[25]

The osteoplant also modulates the expression of genes encoding for collagenic extracellular matrix proteins such as collagen type 1α1 (COL1A1) and collagen type 3α1 (COL3A1). COL1A1 and COL3A1 are significantly down expressed as compared to the control when exposed to the osteoplant, probably because these genes are activated in the late stage of differentiation and are related to extracellular matrix synthesis.

Compared to the levels in the control cells after seven days of treatment, ALPL and FOSL1 were downregulated.

FOSL1 encodes for Fra-1, a component of the dimeric transcription factor activator protein-1 (Ap-1), which is composed mainly of Fos (c-Fos, FosB, Fra-1, and Fra-2) and Jun proteins (c-Jun, JunB, and JunD). AP-1 sites are present in the promoters of many developmentally regulated osteoblast genes, including alkaline phosphatase, collagen I, and osteocalcin. McCabe *et al*.[26] demonstrated that the differential expressions of the Fos and Jun family members could play a role in the developmental regulation of bone-specific gene expression, and as a result, may be functionally significant for osteoblast differentiation. Kim *et al*.[27] studying the effect of the new anabolic agents that stimulate bone formation, find that this gene is activated in the late stage of differentiation, during calcium deposition.

Alkaline phosphatase regulates mineralization of the bone matrix. Several studies have demonstrated that the potency of individual substances to induce alkaline phosphatase varies in a species-dependent manner. Glucocorticoids, such as dexamethasone, are potent inducers in human and rat stromal cells, but they have no effect on alkaline phosphatase activity in mouse stromal cells.[28,29] On the contrary, bone morphogenetic proteins (BMPs) are potent inducers of osteogenesis in both mouse and rat bone marrow stromal cells,[30] but Diefenderfer *et al*. have shown that BMP-2 alone is a poor osteoblast inducer in human marrow-derived stromal cells.[31]

In addition El-Sabban *et al*.[32] demonstrated that Collos, an extract from bovine and equine bone matrix, had opposite effects on osteoblastic differentiation, depending on the differentiated state of the cells. Collos increased proliferation and decreased alkaline phosphatase activity of undifferentiated mesenchymal stem cells and diminished proliferation and increased alkaline phosphatase activity and collagen synthesis of osteoblastic differentiated cells.

The present study shows the effect of osteoplant on PB-hMSCs in the early differentiation stages, as indicated by the activation of bone-related markers RUNX1, BGLAP, and SPP1. The downregulation of genes such as SP7, ALPL, and collagens demonstrated that one week of treatment was not enough for osteoblast differentiation. Moreover, we had chosen to perform the experiment after seven days in order to get information on the early stages of stimulation. It is our understanding, therefore, that more investigations with different time points are needed in order to get a global comprehension of the molecular events related to osteoplant. The reported model is useful to investigate the effects of different substances on stem cells.
